# Examining the relationship between adolescents' social media addiction levels and cyberbullying experiences

**DOI:** 10.1590/1980-220X-REEUSP-2024-0233en

**Published:** 2025-01-13

**Authors:** Sibel Kucuk, Dilek Uludasdemir, Seher Ari Unal

**Affiliations:** 1 Ankara Yildirim Beyazit University, Faculty of Health Sciences, Nursing Department, Ankara, Türkiye.; 2 Manisa Public Hospital, Manisa, Türkiye.

**Keywords:** Adolescente, Ciberacoso, Trastorno de Adicción a Internet, Adolescent, Cyberbullying, Internet Addiction Disorder

## Abstract

**Objective:**

The present study examines the relationship between social media addiction and cyberbullying among adolescents.

**Method:**

This descriptive study was conducted with the participation of 1,058 adolescents aged 14 to 17, between September 1, 2018, and January 1, 2019, in the Central Anatolian region of Türkiye. Data were collected using the Adolescent Data Collection Form, the Revised Cyber Bullying Inventory II, and the Social Media Disorder Scale for Adolescents – Short Form. Data were evaluated with a Pearson’s Chi-square Test, Spearman’s Correlation, and Logistic Regression analysis.

**Results:**

Of the adolescents in the study group, 23.3% were addicted to social media. Female sex and spending more time on social media increased the risk of the development of a social media addiction. Spending more time on social media increased the risk of becoming a victim of cyberbullying and cyber victimization. A strong and positive relationship was identified between social media addiction among adolescents on the one hand, and cyberbullying and victimization on the other.

**Conclusion:**

The more time spent on social media, the greater the risk of development of social media addiction, cyberbullying, and victimization.

## RESUMEN

**Objetivo:**

El presente estudio examina la relación entre la adicción a las redes sociales y el ciberacoso entre adolescentes.

**Método:**

Este estudio descriptivo se realizó con la participación de 1.058 adolescentes de 14 a 17 años, entre el 1 de septiembre de 2018 y el 1 de enero de 2019, en la región de Anatolia Central de Turquía. Los datos se recopilaron utilizando el Formulario de recopilación de datos para adolescentes, el Inventario revisado de acoso cibernético II y la Escala de trastorno de las redes sociales para adolescentes - versión corta. Los datos se evaluaron con una prueba de Chi-cuadrado de Pearson, correlación de Spearman y análisis de regresión logística.

**Resultados:**

Del grupo de estudio, el 23,3% eran adictos a las redes sociales. Ser mujer y pasar más tiempo en las redes sociales aumentó el riesgo de desarrollo de una adicción a las redes sociales. Pasar más tiempo en las redes sociales aumentó el riesgo de convertirse en víctima de acoso y victimización cibernética. Se identificó una relación fuerte y positiva entre la adicción a las redes sociales entre los adolescentes, por un lado, y el acoso cibernético y la victimización, por el otro.

**Conclusión:**

Cuanto más tiempo se pase en las redes sociales, mayor será el riesgo de desarrollar adicción a las redes sociales, acoso cibernético y victimización.

## INTRODUCTION

Paralleling global trends, the use of social media is on the rise also in Türkiye, where 82.4% of people with Internet access have social media accounts, ahead of all other uses of the Internet^(1)^. Social media can be very attractive to adolescents, who have the ability to adapt to change very quickly^(2)^, and who are drawn to it for a wide range of reasons. The Internet and social media have many things to offer adolescents, such as freedom, and opportunities to form their identity and maintain social relationships^(3)^; however, they also give rise to a number of problems.

Excessive Internet use can result in Internet addiction^(4)^, while excessive use of social media can lead to social media addiction^(5)^. Levels of social media addiction among adolescents are higher than in adults^(6)^. Excessive use of social media^(7)^ and the ease at which feelings of anger and animosity can be expressed on social media^(8)^ can have repercussions for adolescents, increasing the risk of engaging in or being subjected to cyberbullying. The problematic use of social media has made cyberbullying and cyber victimization inevitable^(9)^. According to Zhu et al.^(10)^, prevalence rates of cyberbullying have ranged from 6.0% to 46.3%, while cybervictimization rates have ranged from 13.99% to 57.5%. Social media use among adolescents has been reported to double the likelihood of cyber victimization and to increase cyberbullying by six times^(11)^. Cyberbullying, which occurs most frequently through social media accounts, can have negative impacts on adolescents’ psychological and physiological health^(12,13)^.

Social media status as a center of attraction among young people, and the direct and unfiltered consumption of the content offered on social media can have consequences in terms of social media addiction^(8)^, cyberbullying and victimization. It is thus important to determine the relationship, if any, between social media addiction and cyberbullying, which can hamper the development of adolescents into healthy adults. Addressing cyberbullying and social media addiction is still mostly conducted by schools, psychologists, and youth activists^(14)^. However, nurses also have important responsibilities in providing comprehensive nursing care in the prevention and intervention of cyberbullying and social media addiction. Nurses who are in constant relation with children and adolescents should also be involved in determining the changing social media use and cyberbullying characteristics of adolescents living in different regions. Addressing this issue, the present study aims to examine the relationship between social media addiction and cyberbullying among adolescents, thus contributing to the existing literature. Additionally, the relationship between social media use, cyberbullying, and victimization among adolescents in different regions has been evaluated from a nursing perspective.

## METHOD

### Objective and Study Design

This descriptive study^(15)^ examines the relationship between social media addiction and cyberbullying/victimization among adolescents aged 14–17.

### Participants

The universe of the study comprised 10,745 students attending 27 public high schools in a province where the research was conducted in the 2018–2019 academic year (National Education Statistics [NES], 2017). Based on a sample size calculation method for a known population, the minimum sample size required was determined to be 377 (N = 10,745; t = 1.96, P = 0.5; q = 0.5; d = 0.05). To reach this number, schools were categorized based on the type of education they provided, and one school from each of the five categories – general, vocational, Anatolian, science and religious – was selected at random, and one section was then selected randomly from the freshman, sophomore, junior, and senior classes in each of the selected schools. Included in the study were adolescent students attending classes, who had access to the Internet via their computers or cellphones, and who volunteered to participate in the study. The study was conducted with the participation of 1,058 adolescents from five different high schools.

### Data Collection

Data were collected between September 1, 2018 and January 1, 2019 using the Adolescent Data Collection Form, the Revised Cyber Bullying Inventory II (RCBI-II) and the Social Media Disorder Scale for Adolescents – Short Form (SMDSA-SF). The data collection forms were written in Turkish, and were administered over 30 minutes in classroom environments through face-to-face interviews.

Adolescent Data Collection Form: The Adolescent Data Collection Form consisted of a total of eight items garnering data on socio-demographics (age, gender, grade, type of school), mobile phone ownership, social media use, social media accounts, and time spent each day on social media.

Social Media Disorder Scale for Adolescents – Short Form (SMDSA-SF): The Social Media Disorder Scale (SMDS) was developed by Van den Eijnden et al.^(16)^. Tas^(17)^ confirmed the validity and reliability of the nine-item social media disorder scale for use among adolescents in Türkiye. Participants are asked to respond “Yes” or “No” to the items on the scale. Individual items are scored as either 0 or 1, making a total possible score range of 0–9. A cutoff score of 5 has been defined, and those scoring 5 or above are considered to have a social media addiction. In the present study, the internal consistency coefficient of the scale was found to be 0.72.

Revised Cyber Bullying Inventory II (RCBI-II): The validity and reliability of this scale for use in Türkiye were confirmed by Topcu and Erdur-Baker (2018)^(18)^. The scale consists of 10 items that measure the frequency of engagement in or being subjected to cyberbullying by adolescents within the last 6 months. The participants’ scores in the “It happened to me” section measure their experience of victimization, while the scores in the “I did it” section measure their experience of bullying. The participants rate themselves using a four-point Likert scale (1 = never, 2 = once, 3 = twice or three times, 4 = more than three times). The internal consistency coefficient of the scale was found to be 0.84 for the cyber victimization section, and 0.69 for the cyberbullying section. In the present study, the internal consistency coefficient of the scale was found to be 0.81 for the cyberbullying section (I did it) and 0.75 for the cyber victimization section (It happened to me).

### Ethical Considerations

The study was conducted with legal (Kirikkale Provincial Directorate of National Education/8.17.2018/14860805) and ethics permission (Ankara Yildirim Beyazit University Ethics Commite/6.28.2018/08), and informed consent was obtained from the adolescents for their participation. A brief informational session on social media addiction, cyberbullying, and victimization was provided to participants after the data collection process was completed.

### Statistical Analysis

Mean, standard deviation, and median (min, max) values were used for the evaluation of numerical data, while frequencies and percentages were used for the evaluation of categorical data, and Pearson’s Chi-square test was used to examine the effect of one variable on another. Spearman’s Product Moment Correlation was used to examine the relationship between the Social Media Disorder Scale for Adolescents-Short Form (SMDSA-SF) and the Revised Cyber Bullying Inventory II. A preliminary analysis was conducted to check for normality, covariance, and related assumptions. The potential factors affecting cyber victimization, cyberbullying, and social media addiction (those scoring p < 0.05 in univariate analyses) were used to conduct a logistic regression analysis with variables selected to maximize the correct classification percentage (forward or backward). Odds ratios, 95% confidence intervals, Wald statistics and p values are reported. Analyses were conducted using IBM SPSS Statistics for Windows (Version 21.0. Armonk, NY: IBM Corp.), and the level of significance was set at p < 0.05.

## RESULTS

Although not reported in the table, the mean age of the participants was 15.43 ± 1.07 (min = 14; max = 17) and 50.1% were male. Of the total, 87.9% had a mobile phone with an Internet connection, 94.0% had social media accounts, and 48.7% of females and 51.3% of males said they used social media. Among the different social media accounts, Instagram (78.2%) was used most among the respondents, 47.2% make friends through social media with people they do not know, 75.3% share personal photos, and 21.4% share school information. On average, the participants spent 191.00 ± 203.77 (min = 5; max = 1440) minutes a day on social media, and 51.3% stated that they spent more than 3 hours a day on social media. Although not reported in the table, the mean total score from the SMDSA-SF was 2.84 ± 2.28 (Min = 0; Max = 9), with 23.3% of all participants – 26.1% of females and 20.7% of males – being classified as social media addicts.

The difference between the use of Instagram, Snapchat, and Tumblr and social media addiction was statistically significant (p < 0.05). There was a significant relationship between social media addiction and sharing personal information such as school, personal photo, personal video, and birth date (p < 0.001). There was also a significant relationship between social media addiction and being friends with people they do not know, and 31.5% of adolescents who are friends with people they do not know on social media are social media addicts (p < 0.05). Of the respondents who had social media accounts, 24.2% had a social media addiction, and 36.5% of them spent more than 3 hours a day on social media (Table 1).

**Table 1:** Comparison of the Social Media Addiction Scale Short Form for Adolescents (SMAS-SF) and the characteristics of age, gender, social media addiction, and social media use – Kirikkale, CA, Türkiye, 2019.

Features	SMAS-SF		Analysis	
	Addicted	Non addicted		
	n (%)	n (%)	χ^2^	P
**Gender (n = 1058)**			
Female	135 (26.1)	383 (73.9)	4.183	**0.041**
Male	112 (20.7)	428 (79.3)		
**Age (n = 1058)**				
14	62 (24.3)	193 (75.7)	0.281	0.964
15	70 (22.4)	242 (77.6)		
16	62 (23.5)	202 (76.5)		
17	53 (23.3)	174 (76.7)		
**Having a mobile phone connected to the Internet (n = 1058)**				
Yes	225 (24.2)	705 (75.8)	3.086	0.079
No	22 (17.2)	106 (82.8)		
Social media accounts used * (n = 993)				
Facebook	85 (21.5)	311 (78.5)	1.252	0.263
Instagram	217 (26.2)	610 (73.8)	17.720	**0.000**
WhatsApp	168 (24.1)	530 (75.9)	0.599	0.439
Twitter	64 (25.9)	183 (74,1)	1.185	0.276
Youtube	79 (25.2)	234 (74.8)	0.891	0.345
Snapchat	63 (32.8)	129 (67.2)	11.746	**0.001**
Tumblr	20 (38.5)	32 (61.5)	6.982	**0.008**
Tiktok	16 (34.8)	30 (65.2)	3.515	0.061
**Be friends with strangers on social media (n = 993)**				
Yes	149 (31.8)	320 (68.2)	28.014	**0.000**
No	91 (17.4)	433 (82.6)		
**Shared personal information on social media (n = 993)**				
Gender	131 (25.6)	380 (74.4)	2.896	0.089
Birth date	99 (28.1)	253 (71.9)	6.732	**0.009**
School information	70 (31.0)	156 (69.0)	9.343	**0.002**
Personal photograph	210 (26.3)	587 (73.7)	16.279	**0.000**
Personal video	83 (36.6)	144 (63.4)	28.216	**0.000**
Address	11(29.7)	26 (70.3)	0.873	0.350
**Average time spent daily on social media (n = 968)**				
Less than 3 hours	124 (18.5)	544 (81.5)	36.307	**0.000**
3 hours or more	109 (36.5)	191 (63.5)		

*The question has been answered more than once. Percentages are calculated over the numbers of n.

Although not reported in the table, the mean total RCB-II cyberbullying score was 13.36 ± 4.56 (min = 10; max = 40), and the mean total cyber victimization score was 14.05 ± 5.56 (min = 10; max = 40). Of the adolescents who participated in the study, 66.8% were classified as cyberbullies and 64.7% as cyber victims, according to the defined criteria.

It has been determined that 66.1% of the adolescents are cyberbullies and 66.4% are cyber victims who have a mobile phone that is constantly connected to the Internet. There was a statistically significant relationship between the use of Instagram, WhatsApp, Twitter, YouTube, Snapchat and Tumblr social media accounts and cyberbullying and victimization (p < 0.05). Furthermore, a statistically significant relationship was identified between cyber victimization and cyber bullying in terms of sharing of personal information (p < 0.05). Of the adolescents who spent 3 or more hours a day on social media, 83.3% were classified as cyberbullies and 75.6% as cyber victims. There was a statistically significant relationship between the time spent on social media and social media addiction depending on whether the participants had social media accounts (p < 0.05; Table 2).

**Table 2:** Comparison of the Revised Cyber Bullying Inventory-II (RCBI-II) with the characteristics of age, gender, social media addiction, and social media use – Kirikkale, CA, Türkiye, 2019.

Features	Cyberbullying		Analysis*		Cyber victimization		Analysis*	
	Cyber bully	Non-cyberbully	χ^2^	p	Cyber victim	Non-cyber victim	χ^2^	p
	n(%)	n(%)			n(%)	n(%)		
**Gender**							
Male	324 (62.5)	194 (37.5)	1.788	0.181	328 (63.3)	190 (36.7)	0.308	0.579
Female	359 (66.5)	181 (33.5)			333 (61.7)	207 (38.3)		
**Age**							
14	144 (56.5)	111 (43.5)	10.940	**0.012**	149 (58.4)	106 (41.6)	3.675	0.299
15	210 (67.5)	102 (32.7)			206 (66.0)	106 (34.0)		
16	183 (69.3)	81 (30.7)			167 (63.3)	97 (36.7)		
17	146 (64.3)	81 (35.7)			139 (61.2)	88 (38.8)		
**Social media addiction (Based on SMAS-SF Scores)**							
Yes	197 (79.8)	50 (20.2)	32.542	**0.000**	197 (79.8)	50 (20.2)	41.045	**0.000**
No	486 (59.9)	325 (40.1)			464 (57.2)	347 (42.8)		
**Having a mobile phone connected to the Internet (n = 1058)**							
Yes	615 (66.1)	315 (33.9)	8.315	**0.004**	599 (64.4)	331 (35.6)	12.242	**0.000**
No	68 (53.1)	60 (46.9)			62 (48.4)	66 (51.6)		
**Social media accounts used**							
Facebook	266 (67.2)	5 (9.6)	1.893	0.169	246 (62.1)	150 (37.9)	0.034	0.854
Instagram	574 (69.4)	253 (30.6)	38.967	**0.000**	549 (66.4)	278 (33.6)	24.678	**0.000**
WhatsApp	470 (67.3)	228 (32.7)	6.926	**0.008**	464 (66.5)	234 (33.5)	13.995	**0.000**
Twitter	192 (77.2)	55 (22.3)	24.452	**0.000**	180 (72.9)	67 (27.1)	14.861	**0.000**
Youtube	231 (73.8)	82 (26.2)	16.608	**0.000**	226 (72.7)	87 (27.8)	17.944	**0.000**
Snapchat	143 (74.5)	49 (25.5)	10.095	**0.001**	134 (69.8)	58 (30.2)	5.354	**0.021**
Tumblr	47 (90.4)	5 (9.6)	15.945	**0.000**	44 (84.6)	8 (15.4)	11.434	**0.001**
Tiktok	34 (73.9)	12 (26.1)	1.840	0.175	27 (58.7)	19 (41.3)	0.293	0.588
**Be friends with strangers on social media**							
Yes	372 (79.3)	97 (20.7)	64.011	**0.000**	345 (73.6)	124 (26.4)	32.191	**0.000**
No	290 (55.3)	234 (44.7)			295 (56.3)	229 (43.7)		
**Shared personal information on social media**							
Gender	377 (73.8)	134 (26.2)	36.729	**0.000**	353 (69.1)	158 (30.9)	18.386	**0.000**
Birth date	265 (75.3)	87 (24.7)	26.534	**0.000**	241 (68.5)	111 (31.5)	8.072	**0.004**
School information	163 (72.1)	63 (27.9)	7.194	**0.007**	164 (72.6)	62 27.4)	12.481	**0.000**
Personal photograph	554 (69.5)	243 (30.5)	34.665	**0.000**	530 (66.5)	267 (33.5)	22.304	**0.000**
Personal video	178 (78.4)	49 (21.6)	24.258	**0.000**	164 (72.2)	63 (27.8)	11.768	**0.001**
Address	30 (81.1)	7 (18.9)	4.576	**0.032**	23 (62.2)	14 (37.8)	0.002	0.968
**Average time spent daily on social media**							
Less than 3 hours	395 (59.0)	274 (41.0)	54.501	**0.000**	403 (60.2)	266 (39.8)	21.386	**0.000**
3 hours or more	249 (83.3)	50 (16.7)			226 (75.6)	73 (24.4)		

*Statistical significance p < 0.05. Pearson Chi-square test. Row percentages are given.

There were statistically significant gender-based differences in the time spent on social media and social media addiction (p < 0.05). The probability of an adolescent being addicted to social media was 0.74 times higher for males, 0.37 times higher for those with social media accounts, and 2.52 times higher for those who spent three hours or more per day on social media (p < 0.05), according to results of a univariate analysis of the factors determined to have an effect on social media addiction. Adolescents who shared their personal photographs and personal videos on social media and who befriended strangers through their social media accounts were more prone to social media addiction than those who did not (0.5 times and 0.4 times, respectively) (Table 3).

**Table 3:** Univariate logistic regression analysis of features affecting social media addiction – Kirikkale, CA, Türkiye, 2019.

Features	Odds ratio	%95 CI for EXP (B)		Wald test statistics	p*
		Lower	Upper		
**Gender**				
Female/Male	0.742	0.558	0.988	4.169	**0.041**
Constant	0.304			266.899	**0.000**
**Social media accounts used**				
Tumblr	0.583	0.323	1.054	3.192	0.074
Snapchat	0.641	0.451	0.911	6.142	**0.013**
Instagram	0.460	0.302	0.699	13.252	**0.000**
Constant	0.828			0.386	0.534
**Be friends with strangers on social media**				
Yes/No	0.451	0.335	0.608	27.354	**0.000**
Constant	4.66			59.399	**0.000**
**Shared personal information on social media**				
Gender	1.048	0.726	1.512	0.063	0.802
Birth date	0.858	0.584	1.260	0.613	0.434
School information	0.759	0.526	1.095	2.179	0.140
Personal photograph	0.566	0.381	0.840	7.960	**0.005**
Personal video	0.532	0.377	0.751	12.829	**0.000**
Constant	0.773			3.090	0.790
**Time spent on social media in a day**				
Spending more than 3 hours per day on social media/less than 3 hours per day on social media	2.521	1.857	3.423	35.145	**0.000**
Constant	0.228			221.417	**0.000**

*Univariate logistic regression analysis.

A univariate analysis of the factors identified as having an effect on cyberbullying (p < 0.05) revealed that social media addiction, having a mobile phone, befriending strangers on social media accounts, and spending 3 or more hours a day on social media increased the likelihood of cyberbullying by factors of 2.63, 0.58, 3.55, and 3.45, respectively. A univariate analysis of the factors identified as having an effect on cyber victimization (p < 0.05) showed that social media addiction, having a mobile phone, befriending strangers on social media accounts and spending 3 or more hours a day on social media increased the likelihood of cyber victimization by factors of 2.94, 0.51, 0.40, and 2.04, respectively. Adolescents who shared their gender information, personal photographs, and personal videos on social media were more likely to engage in cyberbullying behaviors than those who did not. Furthermore, adolescents who shared their gender and school information and personal videos on social media were more likely to be victims of cyber bullying than those who did not (Table 4).

**Table 4:** Univariate logistic regression analysis of features affecting cyberbullying and victimization – Kirikkale, CA, Türkiye, 2019.

Features	Odds ratio	%95 CI for EXP (B)			Wald test statistics	p*
		Lower	Upper			
***CYBERBULLYING***					
**Social media addicted**					
Social media addicted/non-addicted (Based on SMDS-SF Scores)	2.635	1.874		3.704	31.067	**0.000**
Constant	1.495				31.534	**0.000**
**Having a mobile phone connected to the Internet (n = 1058)**					
Yes/No	0.580	0.400		0.843	8.178	**0.004**
Constant	1.652				44.941	**0.000**
**Social media accounts used**					
Instagram	0.516	0.375		0.709	16.672	**0.000**
WhatsApp	0.887	0.668		1.179	0.681	0.409
Youtube	0.645	0.476		0.874	8.028	**0.005**
Twitter	0.592	0.418		0.838	8.757	**0.003**
Snapchat	0.791	0.545		1.147	1.530	0.216
Tumblr	0.258	0.100		0.665	7.854	**0.005**
Constant	20.792				35.810	0.000
**Shared personal information**					
Gender	0.539	0.390		0.744	14.115	**0.000**
Birth Date	0.763	0.533		1.094	2.163	0.141
School information	1.103	0.764		1.591	0.273	0.601
Personal photograph	0.471	0.348		0.635	24.175	**0.000**
Personal video	0.643	0.443		0.934	5.375	**0.020**
Address	0.676	0.278		1.641	0.750	0.387
Constant	42.384				16.316	**0.000**
**Be friends with strangers on social media account**					
Yes/No	3.551	2.685		4.696	79.019	**0.000**
Constant	2.089				106.829	**0.000**
**Time spent on social media in a day**					
Spending more than 3 hours per day on social media/less than 3 hours per day on social media	3.454	2.457		4.856	50.891	**0.000**
Constant	1.442				21.643	**0.000**
***CYBER VICTIMIZATION***					
**Social media addicted/non-addicted (Based on SMDS-SF Scores)**	2.947	2.097	4.140		38.778	**0.000**
Constant	1.337				16.761	**0.000**
**Having a mobile phone connected to the Internet (n = 1058)**					
Yes/No	0.519	0.358	0.753		11.951	**0.001**
Constant	1.810				75.005	**0.000**
**Social media accounts used**					
Instagram	0.631	0.460	0.865		8.149	**0.004**
WhatsApp	0.719	0.545	0.948		5.476	**0.019**
Twitter	0.624	0.464	0.839		9.751	**0.002**
Youtube	0.687	0.494	0.956		4.960	**0.026**
Snapchat	0.926	0.649	1.321		0.180	0.671
Tumblr	0.363	0.166	0.793		6.454	**0.011**
Constant	11.080				32.480	**0.000**
**Shared personal information**					
Gender	0.634	0.462	0.869		8.023	**0.005**
Birth Date	1.032	0.729	1.460		0.031	0.860
School information	**0.704**	**0.491**	**1.008**		**3.667**	**0.056**
Personal photograph	0.759	0.534	1.077		15.667	0.123
Personal video	0.550	0.409	0.739		2.380	**0.000**
Address	1.686	0.812	3.501		1.961	0.161
Constant	4.753				4.348	**0.037**
**Be friends with strangers on social media account**					
Yes/No	0.401	0.308	0.522		45.833	**0.000**
Constant	1.801				76.049	**0.000**
**Time spent on social media in a day**					
Spending more than 3 hours per day on social media/less than 3 hours per day on social media	2.043	1.505	0.522		20.961	**0.000**
Constant	1.515				27.655	**0.000**

*Univariate logistic regression analysis.

A strong and positive relationship was identified between social media addiction among adolescents and cyberbullying and victimization (Table 5).

**Table 5:** The relationship between social media addiction and cyberbullying and cyber victimization – Kirikkale, CA, Türkiye, 2019.

Variable	Cyber bullying	Cyber victimization	Social Media Addiction
**Cyberbullying**	1	.536*	.170*
**Cyber Victimization**	.536*	1	.191*
**Social Media Addiction**	.170*	.191*	1

*Spearman product moments correlation, p < 0.001.

## DISCUSSION

This study examines the relationships between social media addiction and cyberbullying and cyber victimization among adolescents. In this study, it has been determined that the rate of adolescents’ use of social media is higher than Türkiye’s average given in the OECD 2019 report^(19)^. The rather high prevalence of social media use among the adolescents participating in the present study suggests that the use of social media is becoming more common among the younger population in Türkiye.

Our results demonstrate that the rates of social media addiction among adolescents align with those reported in the literature^(20,21)^. A statistically significant relationship was found between social media addiction and gender in adolescents (p < 0.05) (Table 2). Although the social media usage rates of male adolescents were higher than those of females in the present study (Table 1), female adolescents were 0.74 times more likely to become social media addicts than males (Table 4), which concurs with the findings of earlier studies in the literature. This finding may be related to the fact that females spend more time on social media than males^(22)^.

In the present study, the adolescents stated that they shared personal information with their friends and with people they do not know on social media (Table 1). Those who befriend people they do not know on social media and share personal photographs and videos were found to be more likely to develop social media addiction (0.4 times and 0.5 times more likely, respectively) (Table 4). This finding is consistent with Aljohani et al.^(23)^, who reported that adolescents frequently share personal details on social media. It is apparent that the use of social media constitutes an important part of the daily lives of adolescents, and they share important information about themselves in these environments. The inclination to share personal information may stem from a desire to be part of a larger community^(23)^, which can sometimes overshadow perceived risks.

In the present study, the mean amount of time spent by the respondents on social media (Table 1) concurred with the findings of George et al.^(24)^, but were lower than the OECD average^(19)^. Spending more time on social media is reported to be associated with social media addiction, increasing its risk^(5,20)^, as well as that of cyberbullying and victimization^(15)^. Brannigan et al.^(25)^ state that the ideal daily social media usage time for adolescents should be in the range of 1-2 hours. The present study confirms that having a social media account and spending more time on these platforms significantly increases the risk of social media addiction. Adolescents with social media accounts who spend 3 or more hours a day on them are 2.5 times more likely to develop social media addiction (Table 4). Social media use is an important part of adolescents’ daily lives, and the amount of time spent on them is an important risk factor for the development of social media addiction. An underlying social media addiction may lead to more time being spent on social media, just as spending more time on social media may result in the development of social media addiction. Either way, a strong relationship exists between the time spent on social media and addiction to them (Table 4).

Social media use, as a known risk factor for cyberbullying and victimization, increases the likelihood of engaging in or being subjected to cyberbullying^(21)^, and adolescents who use social media are reportedly more likely to be cyberbullies than non-users^(7,11)^. The present study also revealed a link between social media addiction and possession of a social media account, and cyberbullying and victimization (p < 0.05) (Table 3). The use of social media among adolescents has been reported to increase the risk of cyberbullying by a factor of 6^(11)^, and the risk of cyber victimization by a factor 1.1^(12)^. While the risk of engaging in or being subjected to cyberbullying was found to be lower in the present study than in previous studies, it was still identified as a significant risk. This study showed that almost nine out of 10 adolescents have a mobile phone with a continuous connection to the Internet (Table 1), increasing the risk of cyberbullying and cyber victimization by 0.5 times (Table 3, Table 5).

It was also found that the time spent on social media influenced the rates of cyberbullying and cyber victimization, with adolescents who spend 3 or more hours a day on social media being more likely to engage in cyberbullying (by 3.45 times) or to be exposed to victimization (2.04 times) (Table 5), which concurs with the findings of Taylan et al.^(11)^. Spending more time on social media is thus an important risk factor for cyberbullying and victimization. O’Dea and Campbell (2012) report that adolescents with social media accounts may be more exposed to cyberbullying behaviors such as deception and inappropriate aggressive messages^(26)^. In this study, however, adolescents who make friends with unknown individuals on social media were found to have a significantly higher risk of being cyberbullies (3.5 times) compared to the risk of being cybervictims (0.4 times) (Table 5).

The findings of the present study reveal that social media addiction, along with social media use, increased the likelihood of becoming a cyberbully or victim, and a strong and positive relationship has been identified between social media addiction and cyberbullying (2.63 times) and victimization (2.94 times) (Tables 4 & 5). In parallel with adolescents’ social media addiction, cyberbullying and victimization experiences are also increased. Social media addiction appears to be a significant cause of cyberbullying and cybervictimization for adolescents. Similar studies have identified a positive relationship between social media addiction, and cyberbullying and victimization, and social media addiction has been identified as the most important predictor of cyberbullying^(27,28)^. In short, social media addiction among adolescents is associated with cyberbullying and victimization, being an important risk factor for their occurrence. In parallel with the higher problematic internet use in low-income countries^(29)^, it can be expected that social media addiction and cyberbullying rates will also increase in these countries. Considering that internet use is higher in upper-middle income countries such as Türkiye than in low-income countries, risks are not managed correctly and adolescents may face risks such as social media addiction and cyberbullying. It is thus important to determine the relationship, if any, between social media addiction and cyberbullying, which can hamper the development of adolescents into healthy adults. It was thought that it is important for nurses to take an active role in the management of these risks. For this reason, nurses need to be aware of social media addiction and cyberbullying, which are increasing in frequency with the developing internet technology. Professionals such as teachers who work with adolescents, along with parents, can guide adolescents on the healthy use of digital platforms such as social media, on internet literacy, and cyber risks. For these reasons, school health nurses, in particular, should conduct awareness activities regarding cyber risks, such as social media use, cyberbullying, and cyber victimization for educators, other school staff, and families. In training for families and educators, nurses can address topics such as social media use, use of social media to alleviate negative moods, increased use of social media to improve negative moods, experience of discomfort, irritability, or frustration when not using social media, and the symptoms of social media addiction, including how social media can harm adolescents’ social lives, emotional well-being, academic performance, daily activities, and their careers or other activities and needs^(30)^. Additionality, it is reported that 1-2 hours of daily digital media use is appropriate for adolescents and can have positive effects on symptoms related to digital media usage^(25)^. Therefore, it is crucial for parents to keep their children’s social media usage time at an ideal level. Nurses can inform parents about social media usage time, helping them observe their children’s digital media habits and provide conscious guidance to maintain a healthy balance. By educating parents on the impact of digital media, nurses can play a key role in promoting healthier digital habits for adolescents. Identifying the risks associated with internet use can make it easier to take precautions. It is hoped that this study will guide both pediatric nurses and other professionals working with adolescents in terms of revealing the relationship between social media addiction and cyber bullying. The limitations of the study include the voluntary participation of only 14–17-year-old high-school students. The results obtained from this study were collected using self-report measuring tools reliant on the participants’ perceptions.

## CONCLUSION

This study examined the relationship between social media addiction and cyberbullying/victimization among adolescents aged 14–17. A quarter of the children who participated in the present study reported a social media addiction, while more than half had engaged in or been victims of cyberbullying. When considered together with social media addiction in adolescents, it is observed that the rates of cyberbullying and cyber victimization are quite high. It is seen that both social media addiction and cyberbullying and cyber victimization are important problems for adolescents. Being female, using social media, and spending more time on social media are important risk factors for social media addiction among adolescents, and social media addiction, in turn, is a risk factor for cyberbullying and cyber victimization. Social media addiction in adolescents is associated with cyberbullying and victimization. Additionality, it is essential for nurses to be aware of these risk factors and to implement strategies that mitigate these risks.
